# Supplementary material for: A meta-analysis of plant facilitation in coastal dune systems: responses, regions, and research gaps
Source: PeerJ. 2015 Feb 12;3:e768. doi: 10.7717/peerj.768 (PMC4330909; doi:10.7717/peerj.768)
Supplement: Table S1 [file peerj-03-768-s001.doc]

**Table S1**. List of 32 studies included in systematic review.

| **Study** | **Author and year** | **Journal** | **Country** | **Region** | **Approach** | **Number of cases for Systematic Review** |
| --- | --- | --- | --- | --- | --- | --- |
| 1 | Muhamed et al. 2013 | FOREST ECOLOGY AND MANAGEMENT | France | temperate | experimental | 6 |
| 2 | Doxford et al. 2013 | JOURNAL OF ECOLOGY | Portugal, Spain, France and UK | temperate | experimental | 16 |
| 3 | Le Bagousse-Pinguet et al. 2013 | JOURNAL OF VEGETATION SCIENCE | France | temperate | experimental | 6 |
| 4 | Castanho et al. 2012 | JOURNAL OF VEGETATION SCIENCE | Brazil | tropical | observational | 4 |
| 5 | Santoro et al. 2012 | JOURNAL OF VEGETATION SCIENCE | Italy | temperate | observational | 4 |
| 6 | Garbin et al. 2012 | PERSPECTIVES IN PLANT ECOLOGY EVOLUTION AND SYSTEMATICS | Brazil | tropical | observational | 1 |
| 7 | Cushman et al. 2011 | JOURNAL OF ECOLOGY | USA | temperate | both | 6 |
| 8 | Munoz Valles et al. 2011 | PLANT ECOLOGY | Spain | temperate | observational | 2 |
| 9 | Grau et al. 2010 | OIKOS | Finland | artic | observational | 3 |
| 10 | Cushman et al. 2010 | JOURNAL OF VEGETATION SCIENCE | USA | temperate | observational | 22 |
| 11 | Maltez-Mouro et al. 2010 | ACTA OECOLOGICA-INTERNATIONAL | Portugal | temperate | observational | 8 |
| 12 | Forey et al. 2010 | PLANT ECOLOGY | France | temperate | experimental | 32 |
| 13 | Forey et al. 2009 | JOURNAL OF VEGETATION SCIENCE | France | temperate | observational | 33 |
| 14 | Armas and Pugnaire 2009 | JOURNAL OF VEGETATION SCIENCE | Spain | temperate | both | 27 |
| 15 | Brancalion et al. 2009 | BIOTROPICA | Brazil | tropical | observational | 1 |
| 16 | Bonanomi et al. 2009 | PLANT ECOLOGY | Italy | temperate | observational | 9 |
| 17 | Feagin and Wu 2007 | RANGELAND ECOLOGY & MANAGEMENT | USA | temperate | observational | 5 |
| 18 | Cheplick 2005 | PLANT ECOLOGY | USA | temperate | observational | 10 |
| 19 | Dias et al. 2005 | JOURNAL OF TROPICAL ECOLOGY | Brazil | tropical | both | 15 |
| 20 | Martinez et al. 2004 | ECOSCIENCE | Mexico | tropical | both | 11 |
| 21 | Sternberg et al. 2004 | ISRAEL JOURNAL OF PLANT SCIENCES | Israel | temperate | observational | 4 |
| 22 | Franks 2003 | PLANT ECOLOGY | USA | tropical | experimental | 1 |
| 23 | Franks and Peterson 2003 | PLANT ECOLOGY | USA | tropical | experimental | 4 |
| 24 | Martinez 2003 | PLANT ECOLOGY | Mexico | tropical | both | 5 |
| 25 | Rudgers and Maron 2003 | OIKOS | USA | temperate | both | 12 |
| 26 | Franks 2003 | CANADIAN JOURNAL OF BOTANY | USA | tropical | experimental | 43 |
| 27 | El-Bana et al. | PLANT AND SOIL | Egito | temperate | observational | 15 |
| 28 | Joy and Young 2002 | PLANT ECOLOGY | USA | temperate | both | 8 |
| 29 | De Villiers et al. 2001 | JOURNAL OF ARID ENVIRONMENTS | South Africa | temperate | both | 25 |
| 30 | Gagne and Houle 2001 | CANADIAN JOURNAL OF BOTANY | Canada | subartic | observational | 5 |
| 31 | Shumway 2000 | OECOLOGIA | USA | temperate | both | 11 |
| 32 | Houle 1997 | CANADIAN JOURNAL OF BOTANY | Canada | subartic | experimental | 8 |
