# Supplementary material for: A meta-analysis of plant facilitation in coastal dune systems: responses, regions, and research gaps
Source: PeerJ. 2015 Feb 12;3:e768. doi: 10.7717/peerj.768 (PMC4330909; doi:10.7717/peerj.768)
Supplement: Table S2 [file peerj-03-768-s002.doc]

**Table S2.** List of 40 cases (outcomes) included in the meta-analysis of density data.

| **Case** | **Author and year** | **Region** | **MAP (mm)** | **Local NDVI** | **Regional NDVI** | **part of the gradient** | **Neighbor life-form** | **Neighbor species** | **Target life-form** | **Target species** | **Target life stage** | **Effect Size (lnRR)** | **Variance (lnRR)** |
| --- | --- | --- | --- | --- | --- | --- | --- | --- | --- | --- | --- | --- | --- |
| 1 | Grau et al. 2010 | artic | 403 | NA | 5.3 | early dune | shrub | *Empetrum nigrum ssp. hermaphroditum* | tree | *Pinus sylvestris* | young | 4.738 | 3631.11 |
| 2 | Grau et al. 2010 | artic | 403 | NA | 5.3 | mid dune | shrub | *Empetrum nigrum ssp. hermaphroditum* | tree | *Pinus sylvestris* | young | 1.064 | 0.189 |
| 3 | Cushman et al. 2010 | temperate | 482 | 0.65 | 4.2 | hind-dune | shrub | *Lupinus chamissonis* | forb | *Claytonia perfoliata* | several | 4.297 | 0.165 |
| 4 | Cushman et al. 2010 | temperate | 482 | 0.65 | 4.2 | hind-dune | shrub | *Ericameria ericoides* | forb | *Claytonia perfoliata* | several | 4.099 | 0.161 |
| 5 | Cushman et al. 2010 | temperate | 482 | 0.65 | 4.2 | hind-dune | shrub | *Lupinus chamissonis* | grass | *Bromus diadruns* | several | 0.627 | 0.296 |
| 6 | Cushman et al. 2010 | temperate | 482 | 0.65 | 4.2 | hind-dune | shrub | *Ericameria ericoides* | grass | *Bromus diadruns* | several | 0.319 | 0.330 |
| 7 | Cushman et al. 2010 | temperate | 482 | 0.65 | 4.2 | hind-dune | shrub | *Lupinus chamissonis* | grass | *Vulpia bromoides* | several | -2.860 | 2.271 |
| 8 | Cushman et al. 2010 | temperate | 482 | 0.65 | 4.2 | hind-dune | shrub | *Ericameria ericoides* | grass | *Vulpia bromoides* | several | -0.915 | 0.119 |
| 9 | Cushman et al. 2010 | temperate | 482 | 0.65 | 4.2 | hind-dune | shrub | *Lupinus chamissonis* | moss | several | several | -2.005 | 0.168 |
| 10 | Cushman et al. 2010 | temperate | 482 | 0.65 | 4.2 | hind-dune | shrub | *Ericameria ericoides* | moss | several | several | 0.836 | 0.046 |
| 11 | Forey et al. 2009 | temperate | 749 | 0.30 | 7.6 | foredune | grass | *Elymus farctus* | several | several | several | 1.748 | 0.260 |
| 12 | Forey et al. 2009 | temperate | 772 | 0.216 | 7.7 | foredune | grass | *Elymus farctus* | several | several | several | 0.742 | 0.219 |
| 13 | Forey et al. 2009 | temperate | 699 | NA | 6.5 | foredune | grass | *Elymus farctus* | several | several | several | -0.045 | 0.105 |
| 14 | Forey et al. 2009 | temperate | 749 | 0.304 | 7.6 | whitedune | grass | *Ammophila arenaria* | several | several | several | 0.064 | 0.110 |
| 15 | Forey et al. 2009 | temperate | 772 | 0.216 | 7.7 | whitedune | grass | *Ammophila arenaria* | several | several | several | 0.541 | 0.074 |
| 16 | Forey et al. 2009 | temperate | 699 | NA | 6.5 | whitedune | grass | *Ammophila arenaria* | several | several | several | 0.126 | 0.024 |
| 17 | Forey et al. 2009 | temperate | 749 | 0.304 | 7.6 | greydune | forb | *Helichrysum stoechas* | several | several | several | 0.136 | 0.020 |
| 18 | Forey et al. 2009 | temperate | 772 | 0.216 | 7.7 | greydune | forb | *Helichrysum stoechas* | several | several | several | 0.340 | 0.016 |
| 19 | Forey et al. 2009 | temperate | 699 | NA | 6.5 | greydune | forb | *Helichrysum stoechas* | several | several | several | -0.319 | 0.017 |
| 20 | Forey et al. 2009 | temperate | 749 | 0.304 | 7.6 | greydune | shrub | *Ephedra distachya* | several | several | several | 0.457 | 0.011 |
| 21 | Forey et al. 2009 | temperate | 699 | NA | 6.5 | greydune | forb | *Pancratium maritimum* | several | several | several | -0.243 | 0.017 |
| 22 | Bonanomi et al. 2008 | temperate | 737 | 0.264 | 6.0 | open shrub | shrub | *Medicago marina* | grass | *Lophochloa pubescens* | several | 1.386 | 0.184 |
| 23 | Cheplick 2005 | temperate | 1874 | NA | 8.4 | pioneer zone | grass | *Ammophila breviligulata* | grass | *Triplasis purpurea* | several | -0.065 | 0.011 |
| 24 | Cheplick 2005 | temperate | 1874 | NA | 8.4 | bpioneer zone | grass | *Ammophila breviligulata* | grass | *Cenchrus tribuloides* | several | -0.571 | 0.010 |
| 25 | Cheplick 2005 | temperate | 1874 | NA | 8.4 | pioneer zone | grass | *Ammophila breviligulata* | forb | *Heterotheca subaxillaris* | several | -0.819 | 0.097 |
| 26 | Martinez et al. 2004 | tropical | 657 | 0.151 | 6.7 | parabolic mobile dunes | shrub | *Chamaecrista chamaecristoides var. chamaecristoides* | grass | *Trachypogon plumosus* | adult | 2.754 | 0.372 |
| 27 | Martinez et al. 2004 | tropical | 657 | 0.151 | 6.7 | parabolic mobile dunes | shrub | *Chamaecrista chamaecristoides var. chamaecristoides* | grass | *Schizachyrium scoparium var. littoralis* | adult | 0.000 | 0.404 |
| 28 | Martinez et al. 2004 | tropical | 657 | 0.151 | 6.7 | parabolic mobile dunes | shrub | *Chamaecrista chamaecristoides var. chamaecristoides* | grass | *Trachypogon plumosus* and *Schizachyrium scoparium var. littoralis* | young | -1.315 | 0.168 |
| 29 | Martinez et al. 2004 | tropical | 657 | 0.151 | 6.7 | parabolic mobile dunes | shrub | *Chamaecrista chamaecristoides var. chamaecristoides* | grass | *Trachypogon plumosus* | adult | 1.689 | 0.254 |
| 30 | Martinez et al. 2004 | tropical | 657 | 0.151 | 6.7 | parabolic mobile dunes | shrub | *Chamaecrista chamaecristoides var. chamaecristoides* | grass | *Schizachyrium scoparium var. littoralis* | adult | -2.019 | 0.116 |
| 31 | Martinez et al. 2004 | tropical | 657 | 0.151 | 6.7 | parabolic mobile dunes | shrub | *Chamaecrista chamaecristoides var. chamaecristoides* | grass | *Trachypogon plumosus* and *Schizachyrium scoparium var. littoralis* | young | -0.644 | 0.266 |
| 32 | Sternberg et al. 2004 | temperate | 347 | NA | 4.4 | open shrub | shrub | *Retama raetam* | herb | several | young | -0.934 | 0.027 |
| 33 | Sternberg et al. 2004 | temperate | 347 | NA | 4.4 | open shrub | shrub | *Retama raetam* | herb | several | several | -2.020 | 0.058 |
| 34 | Joy and Young 2002 | temperate | 958 | 0.256 | 6.9 | open shrub | tree | *Juniperus virginiana* | several | several | several | -0.408 | 0.208 |
| 35 | Joy and Young 2002 | temperate | 958 | 0.256 | 6.9 | open shrub | tree | *Juniperus virginiana* | several | several | several | 0.640 | 0.091 |
| 36 | Gagne and Houle 2001 | subartic | 680 | 0.116 | 3.3 | embryo dunes | forb | *Honckenya peploides* | grass | *Leymus mollis* | young | 3.058 | 0.400 |
| 37 | Shumway 2000 | temperate | 1049 | 0.114 | 7.1 | open shrub | shrub | *Myrica pensylvanica* | grass | *Ammophila breviligulata* | adult | 0.207 | 0.010 |
| 38 | Shumway 2000 | temperate | 1049 | 0.114 | 7.1 | open shrub | shrub | *Myrica pensylvanica* | forb | *Solidago sempervirens* | adult | -0.161 | 0.048 |
| 39 | Shumway 2000 | temperate | 1049 | 0.114 | 7.1 | open shrub | shrub | *Myrica pensylvanica* | grass | *Ammophila breviligulata* | young | -0.317 | 0.579 |
| 40 | Shumway 2000 | temperate | 1049 | 0.114 | 7.1 | open shrub | shrub | *Myrica pensylvanica* | forb | *Solidago sempervirens* | young | 4.928 | 0.399 |

*(Appendix 2 – continued)*
