# Supplementary material for: A meta-analysis of plant facilitation in coastal dune systems: responses, regions, and research gaps
Source: PeerJ. 2015 Feb 12;3:e768. doi: 10.7717/peerj.768 (PMC4330909; doi:10.7717/peerj.768)
Supplement: Table S3 [file peerj-03-768-s003.doc]

**Table S3**. List of 39 cases (outcomes) included in the meta-analysis of growth data.

| **Case** | **Author and year** | **Region** | **MAP (mm)** | **local NDVI** | **regional NDVI** | **part of the gradient** | **Neighbor life-form** | **Neighbor species** | **Target life-form** | **Target species** | **Target life stage** | **Effect Size (lnRR)** | **Variance (lnRR)** |
| --- | --- | --- | --- | --- | --- | --- | --- | --- | --- | --- | --- | --- | --- |
| 1 | Cushman et al. 2011 | temperate | 482 | NA | 4.2 | foredune | grass | *Ammophila arenaria* | grass | *Ehrharta calycina* | adult | 0.725 | 0.216 |
| 2 | Cushman et al. 2011 | temperate | 482 | NA | 4.2 | foredune | shrub | *Baccharis pilularis* | grass | *Ehrharta calycina* | adult | 1.706 | 0.168 |
| 3 | Cushman et al. 2011 | temperate | 482 | NA | 4.2 | foredune | forb | *Carpobrotus edulis* | grass | *Ehrharta calycina* | adult | 0.036 | 0.135 |
| 4 | Cushman et al. 2010 | temperate | 482 | 0.652 | 4.2 | hind-dune | shrub | *Lupinus chamissonis* | grass | several | several | 0.538 | 0.091 |
| 5 | Cushman et al. 2010 | temperate | 482 | 0.652 | 4.2 | hind-dune | shrub | *Ericameria ericoides* | grass | several | several | 0.385 | 0.086 |
| 6 | Cushman et al. 2010 | temperate | 482 | 0.652 | 4.2 | hind-dune | shrub | *Lupinus chamissonis* | forb | several | several | -0.480 | 0.127 |
| 7 | Cushman et al. 2010 | temperate | 482 | 0.652 | 4.2 | hind-dune | shrub | *Ericameria ericoides* | forb | several | several | -0.728 | 0.229 |
| 8 | Forey et al. 2010 | temperate | 772 | NA | 7.7 | foredune | grass | *Elymus farctus* | grass | *Elymus farctus* | young | 0.728 | 0.182 |
| 9 | Forey et al. 2010 | temperate | 772 | NA | 7.7 | foredune | grass | *Elymus farctus* | grass | *Ammophila arenaria* | young | 2.838 | 16.823 |
| 10 | Forey et al. 2010 | temperate | 772 | NA | 7.7 | whitedune | grass | *Ammophila arenaria* | grass | *Elymus farctus* | young | 2.539 | 8.509 |
| 11 | Forey et al. 2010 | temperate | 772 | NA | 7.7 | whitedune | grass | *Ammophila arenaria* | grass | *Ammophila arenaria* | young | 2.781 | 6.255 |
| 12 | Forey et al. 2010 | temperate | 772 | NA | 7.7 | whitedune | grass | *Ammophila arenaria* | forb | *Helichrysum stoechas* | young | 0.030 | 0.234 |
| 13 | Forey et al. 2010 | temperate | 772 | NA | 7.7 | whitedune | grass | *Ammophila arenaria* | grass | *Corynephorus canescens* | young | -0.396 | 0.668 |
| 14 | Forey et al. 2010 | temperate | 772 | NA | 7.7 | transition dune | forb | *Helichrysum stoechas* | grass | *Elymus farctus* | young | 0.162 | 0.647 |
| 15 | Forey et al. 2010 | temperate | 772 | NA | 7.7 | transition dune | forb | *Helichrysum stoechas* | grass | *Ammophila arenaria* | young | 2.890 | 7.225 |
| 16 | Forey et al. 2010 | temperate | 772 | NA | 7.7 | transition dune | forb | *Helichrysum stoechas* | forb | *Helichrysum stoechas* | young | 0.599 | 0.965 |
| 17 | Forey et al. 2010 | temperate | 772 | NA | 7.7 | transition dune | forb | *Helichrysum stoechas* | grass | *Corynephorus canescens* | young | 0.301 | 0.252 |
| 18 | Forey et al. 2010 | temperate | 772 | NA | 7.7 | grey dune | grass | *Corynephorus canescens* | grass | *Elymus farctus* | young | 0.662 | 0.705 |
| 19 | Forey et al. 2010 | temperate | 772 | NA | 7.7 | grey dune | grass | *Corynephorus canescens* | grass | *Ammophila arenaria* | young | 2.825 | 3.206 |
| 20 | Forey et al. 2010 | temperate | 772 | NA | 7.7 | grey dune | grass | *Corynephorus canescens* | forb | *Helichrysum stoechas* | young | -0.350 | 0.272 |
| 21 | Forey et al. 2010 | temperate | 772 | NA | 7.7 | grey dune | grass | *Corynephorus canescens* | grass | *Corynephorus canescens* | young | 0.007 | 0.310 |
| 22 | Armas and Pugnaire 2009 | temperate | 313 | 0.208 | 2.5 | open shrub | shrub | Several | shrub | *Juniperus phoenicea subsp. turbinata* | adult | 0.223 | 0.008 |
| 23 | Armas and Pugnaire 2009 | temperate | 313 | 0.208 | 2.5 | open shrub | shrub | Several | shrub | *Pistacia lentiscus* | adult | 0.088 | 0.005 |
| 24 | Bonanomi et al. 2008 | temperate | 737 | 0.264 | 6.0 | open shrub | shrub | *Medicago marina* | grass | *Lophochloa pubescens* | adult | 1.705 | 0.151 |
| 25 | Sternberg et al. 2004 | temperate | 347 | NA | 4.4 | open shrub | shrub | *Retama raetam* | herb | several | several | -1.365 | 0.103 |
| 26 | Rudgers and Maron 2003 | temperate | 482 | 0.652 | 5.0 | open shrub | shrub | *Baccharis pilularis pilularis* | shrub | *Lupinus arboreus* | young | 0.246 | 0.013 |
| 27 | Rudgers and Maron 2003 | temperate | 482 | 0.652 | 5.0 | open shrub | grass | *Ammophila arenaria* | shrub | *Lupinus arboreus* | young | 0.379 | 0.013 |
| 28 | Franks 2003 | tropical | 1116 | NA | 5.9 | foredune, mid-dune and rear dune | grass | *Uniola paniculata* | grass | *Uniola paniculata* | young | 1.806 | 0.247 |
| 29 | Franks 2003 | tropical | 1116 | NA | 5.9 | foredune, mid-dune and rear dune | shrub | *Iva imbricata* | grass | *Uniola paniculata* | young | 2.517 | 0.406 |
| 30 | Franks 2003 | temperate | 881 | 0.193 | 8.0 | foredune, mid-dune and rear dune | grass | *Uniola paniculata* | grass | *Uniola paniculata* | young | -1.053 | 0.126 |
| 31 | Franks 2003 | temperate | 881 | 0.193 | 8.0 | foredune, mid-dune and rear dune | shrub | *Iva imbricata* | grass | *Uniola paniculata* | young | -1.925 | 0.862 |
| 32 | Franks 2003 | tropical | 1116 | NA | 5.9 | foredune, mid-dune and rear dune | grass | *Uniola paniculata* | shrub | *Iva imbricata* | young | -0.024 | 3.548 |
| 33 | Franks 2003 | tropical | 1116 | NA | 5.9 | foredune, mid-dune and rear dune | shrub | *Iva imbricata* | shrub | *Iva imbricata* | young | 0.915 | 1.093 |
| 34 | Franks 2003 | temperate | 881 | 0.193 | 8.0 | foredune, mid-dune and rear dune | grass | *Uniola paniculata* | shrub | *Iva imbricata* | young | -0.659 | 0.085 |
| 35 | Franks 2003 | temperate | 881 | 0.193 | 8.0 | foredune, mid-dune and rear dune | shrub | *Iva imbricata* | shrub | *Iva imbricata* | young | -0.733 | 0.078 |
| 36 | Gagne and Houle 2001 | subartic | 680 | 0.116 | 3.3 | embryo dunes | forb | *Honckenya peploides* | grass | *Leymus mollis* | young | -0.034 | 0.005 |
| 37 | Shumway 2000 | temperate | 1049 | 0.114 | 7.1 | primary dunes (open shrub) | shrub | *Myrica pensylvanica* | grass | *Ammophila breviligulata* | adult | 0.775 | 0.035 |
| 38 | Shumway 2000 | temperate | 1049 | 0.114 | 7.1 | primary dunes (open shrub) | shrub | *Myrica pensylvanica* | forb | *Solidago sempervirens* | adult | 1.363 | 0.046 |
| 39 | Shumway 2000 | temperate | 1049 | 0.114 | 7.1 | primary dunes (open shrub) | shrub | *Myrica pensylvanica* | forb | *Solidago sempervirens* | adult | 0.365 | 0.014 |

*(Appendix 3 – continued)*
