# Supplementary material for: A meta-analysis of plant facilitation in coastal dune systems: responses, regions, and research gaps
Source: PeerJ. 2015 Feb 12;3:e768. doi: 10.7717/peerj.768 (PMC4330909; doi:10.7717/peerj.768)
Supplement: Table S4 [file peerj-03-768-s004.doc]

**Table S4.** List of 42 cases (outcomes) included in the meta-analysis of survival data.

| **Case** | **Author and year** | **Region** | **MAP (mm)** | **local NDVI** | **regional NDVI** | **part of the gradient** | **Neighbor life-form** | **Neighbor species** | **Target life-form** | **Target species** | **Target life phase** | **Effect Size (lnOR)** | **Variance (lnOR)** |
| --- | --- | --- | --- | --- | --- | --- | --- | --- | --- | --- | --- | --- | --- |
| 1 | Muhamed et al. 2013 | temperate | 712 | NA | 7.7 | forest | tree | *Quercus ilex* or *Quercus robur* | tree | *Quercus robur* | young | 0.979 | 1.327 |
| 2 | Muhamed et al. 2013 | temperate | 712 | NA | 7.7 | forest | tree | *Quercus ilex* or *Quercus robur* | tree | *Quercus ilex* | young | 1.373 | 1.366 |
| 3 | Muhamed et al. 2013 | temperate | 712 | NA | 7.7 | forest | tree | *Quercus ilex* or *Quercus robur* | tree | *Quercus suber* | young | -0.966 | 1.326 |
| 4 | Muhamed et al. 2013 | temperate | 699 | NA | 6.6 | forest | tree | *Quercus suber* or *Ilex aquifolium* | tree | *Quercus robur* | young | -1.358 | 1.364 |
| 5 | Muhamed et al. 2013 | temperate | 699 | NA | 6.6 | forest | tree | *Quercus suber* or *Ilex aquifolium* | tree | *Quercus ilex* | young | -1.694 | 1.407 |
| 6 | Muhamed et al. 2013 | temperate | 699 | NA | 6.6 | forest | tree | *Quercus suber* or *Ilex aquifolium* | tree | *Quercus suber* | young | -6.071 | 2.822 |
| 7 | Le Bagousse-Pinguet et al. 2013 | temperate | 772 | 0.22 | 7.7 | greydunes | forb | *Helichrysum stoechas* | grass | *Festuca vasconensis* | not informed | 0.000 | 1.072 |
| 8 | Le Bagousse-Pinguet et al. 2013 | temperate | 772 | 0.22 | 7.7 | greydunes | forb | *Helichrysum stoechas* | forb | *Senecio inaequidens* | not informed | 0.940 | 1.104 |
| 9 | Le Bagousse-Pinguet et al. 2013 | temperate | 772 | 0.22 | 7.7 | greydunes | forb | *Helichrysum stoechas* | forb | *Pancratium maritimum* | not informed | 0.229 | 1.074 |
| 10 | Le Bagousse-Pinguet et al. 2013 | temperate | 772 | 0.22 | 7.7 | greydunes | forb | *Helichrysum stoechas* | shrub | *Cistus salviifolius* | not informed | 1.211 | 1.125 |
| 11 | Le Bagousse-Pinguet et al. 2013 | temperate | 772 | 0.22 | 7.7 | greydunes | forb | *Helichrysum stoechas* | grass | *Elymus farctus* | not informed | 0.871 | 1.099 |
| 12 | Le Bagousse-Pinguet et al. 2013 | temperate | 772 | 0.22 | 7.7 | greydunes | forb | *Helichrysum stoechas* | forb | *Oenothera biennis* | not informed | 0.814 | 1.096 |
| 13 | Forey et al. 2010 | temperate | 772 | 0.22 | 7.7 | foredune | grass | *Elymus farctus* | grass | *Elymus farctus* | young | -0.345 | 0.690 |
| 14 | Forey et al. 2010 | temperate | 772 | 0.22 | 7.7 | foredune | grass | *Elymus farctus* | grass | *Ammophila arenaria* | young | -0.345 | 0.690 |
| 15 | Forey et al. 2010 | temperate | 772 | 0.22 | 7.7 | foredune | grass | *Elymus farctus* | forb | *Helichrysum stoechas* | young | 0.000 | 4.000 |
| 16 | Forey et al. 2010 | temperate | 772 | 0.22 | 7.7 | foredune | grass | *Elymus farctus* | grass | *Corynephorus canescens* | young | 0.000 | 4.000 |
| 17 | Forey et al. 2010 | temperate | 772 | 0.22 | 7.7 | whitedune | grass | *Ammophila arenaria* | grass | *Elymus farctus* | young | 1.818 | 0.455 |
| 18 | Forey et al. 2010 | temperate | 772 | 0.22 | 7.7 | whitedune | grass | *Ammophila arenaria* | grass | *Ammophila arenaria* | young | 0.667 | 0.444 |
| 19 | Forey et al. 2010 | temperate | 772 | 0.22 | 7.7 | whitedune | grass | *Ammophila arenaria* | forb | *Helichrysum stoechas* | young | 0.486 | 0.486 |
| 20 | Forey et al. 2010 | temperate | 772 | 0.22 | 7.7 | whitedune | grass | *Ammophila arenaria* | grass | *Corynephorus canescens* | young | 0.625 | 0.625 |
| 21 | Forey et al. 2010 | temperate | 772 | 0.22 | 7.7 | transition dune | forb | *Helichrysum stoechas* | grass | *Elymus farctus* | young | 0.909 | 0.455 |
| 22 | Forey et al. 2010 | temperate | 772 | 0.22 | 7.7 | transition dune | forb | *Helichrysum stoechas* | grass | *Ammophila arenaria* | young | 0.000 | 0.438 |
| 23 | Forey et al. 2010 | temperate | 772 | 0.22 | 7.7 | transition dune | forb | *Helichrysum stoechas* | forb | *Helichrysum stoechas* | young | -1.273 | 0.509 |
| 24 | Forey et al. 2010 | temperate | 772 | 0.22 | 7.7 | transition dune | forb | *Helichrysum stoechas* | grass | *Corynephorus canescens* | young | -0.539 | 0.539 |
| 25 | Forey et al. 2010 | temperate | 772 | 0.22 | 7.7 | grey dune | grass | *Corynephorus canescens* | grass | *Elymus farctus* | young | 1.111 | 0.444 |
| 26 | Forey et al. 2010 | temperate | 772 | 0.22 | 7.7 | grey dune | grass | *Corynephorus canescens* | grass | *Ammophila arenaria* | young | -0.455 | 0.455 |
| 27 | Forey et al. 2010 | temperate | 772 | 0.22 | 7.7 | grey dune | grass | *Corynephorus canescens* | forb | *Helichrysum stoechas* | young | -0.217 | 0.433 |
| 28 | Forey et al. 2010 | temperate | 772 | 0.22 | 7.7 | grey dune | grass | *Corynephorus canescens* | grass | *Corynephorus canescens* | young | -0.345 | 0.690 |
| 29 | Armas and Pugnaire 2009 | temperate | 313 | 0.21 | 2.5 | open shrub | shrub | *Juniperus phoenicea subsp. turbinata* | shrub | *Juniperus phoenicea subsp. turbinata* | young | 1.833 | 0.282 |
| 30 | Armas and Pugnaire 2009 | temperate | 313 | 0.21 | 2.5 | open shrub | shrub | *Juniperus phoenicea subsp. turbinata* | shrub | *Pistacia lentiscus* | young | 2.438 | 0.406 |
| 31 | Armas and Pugnaire 2009 | temperate | 313 | 0.21 | 2.5 | open shrub | shrub | *Pistacia lentiscus* | shrub | *Juniperus phoenicea subsp. turbinata* | young | 1.671 | 0.304 |
| 32 | Armas and Pugnaire 2009 | temperate | 313 | 0.21 | 2.5 | open shrub | shrub | *Pistacia lentiscus* | shrub | *Pistacia lentiscus* | young | 1.219 | 0.609 |
| 33 | Armas and Pugnaire 2009 | temperate | 313 | 0.21 | 2.5 | open shrub | shrub | several | shrub | *Juniperus phoenicea subsp. turbinata* | adult | -1.229 | 0.028 |
| 34 | Armas and Pugnaire 2009 | temperate | 313 | 0.21 | 2.50 | open shrub | shrub | several | shrub | *Pistacia lentiscus* | adult | -0.051 | 0.563 |
| 35 | Martinez 2003 | tropical | 657 | 0.15 | 6.7 | parabolic mobile dunes | shrub | *Chamaecrista chamaecristoides var. chamaecristoides* | grass | *Trachypogon plumosus* | young | 2.073 | 0.276 |
| 36 | Martinez 2003 | tropical | 657 | 0.15 | 6.7 | parabolic mobile dunes | shrub | *Chamaecrista chamaecristoides var. chamaecristoides* | grass | *Schizachyrium scoparium var. littoralis* | young | 2.046 | 0.409 |
| 37 | Rudgers and Maron 2003 | temperate | 482 | 0.65 | 5.0 | open shrub | shrub | *Baccharis pilularis pilularis* | shrub | *Lupinus arboreus* | young | 0.683 | 0.085 |
| 38 | Rudgers and Maron 2003 | temperate | 482 | 0.65 | 5.0 | open shrub | grass | *Ammophila arenaria* | shrub | *Lupinus arboreus* | young | -1.119 | 0.124 |
| 39 | Joy and Young 2002 | temperate | 958 | 0.26 | 6.9 | open shrub | tree | *Juniperus virginiana* | tree | *Sassafras albidum* | young | 0.000 | 4.000 |
| 40 | Joy and Young 2002 | temperate | 958 | 0.26 | 6.9 | open shrub | tree | *Juniperus virginiana* | tree | *Prunus serotina* | young | 1.555 | 0.222 |
| 41 | Shumway 2000 | temperate | 1049 | 0.11 | 7.1 | open shrub | shrub | *Myrica pensylvanica* | grass | *Ammophila breviligulata* | young | -0.442 | 0.221 |
| 42 | Shumway 2000 | temperate | 1049 | 0.11 | 7.1 | open shrub | shrub | *Myrica pensylvanica* | forb | *Solidago sempervirens* | young | 0.059 | 0.118 |

*(Appendix 4 – continued)*
