# Supplementary material for: A meta-analysis of plant facilitation in coastal dune systems: responses, regions, and research gaps
Source: PeerJ. 2015 Feb 12;3:e768. doi: 10.7717/peerj.768 (PMC4330909; doi:10.7717/peerj.768)
Supplement: Appendix S1 [file peerj-03-768-s006.doc]

**Appendix S1** References included in Tables S1-S4 and not cited in the main text.

Bonanomi G, Rietkerk M, Dekker SC, Mazzoleni S. 2008. Islands of fertility induce co-occurring negative and positive plant-soil feedbacks promoting coexistence. *Plant Ecology* 197:207-218.

Brancalion PHS, Gabriel VA, Gómez JM . 2009. Do terrestrial tank bromeliads in Brazil create safe sites for palm establishment or act as natural traps for its dispersed seeds? *Biotropica* 41:3-6.

Cheplick GP. 2005. Patterns in the distribution of American beachgrass (*Ammophila breviligulata*) and the density and reproduction of annual plants on a coastal beach. *Plant Ecology* 180: 57-67.

Dias ATC, Zaluar HLT, Ganade G, Scarano FR. 2005. Canopy composition influencing plant patch dynamics in a Brazilian sandy coastal plain. *Journal of Tropical Ecology* 21:343-347.

Doxford, S. W. et al. 2013. Spatial and temporal variability in positive and negative plant-bryophyte interactions along a latitudinal gradient. *Journal of Ecology* 101: 465-474.

El-Bana MI, Nijs I, Kockelbergh F. 2002. Microenvironment and vegetational heterogeneity induced by phytogenic nebkas in a arid coastal ecosystem. *Plant and Soil* 247:283-293.

Feagin RA, Wu XB. 2007. The spatial patterns of functional groups and successional direction in a coastal dune community. *Rangeland Ecology Management* 60:417-425.

Feagin RA, Wu XB, Smeins FE, Whisenant SG, Grant WE . 2005. Individual versus community level processes and pattern formation in a model of sand dune plant sucession. *Ecological Modelling* 183:435-449.

Forey E, Touzard B, Michalet R. 2010. Does disturbance drive the collapse of biotic interactions at the severe end of a diversity-biomass gradient? *Plant Ecology* 206: 287-295.

Franks SJ. 2003. Facilitation in multiple life-history stages: evidence for nucleated succession in coastal dunes. *Plant Ecology* 168:1-11.

Franks SJ. 2003. Competitive and facilitative interactions within and between two species of coastal dune perennials. *Canadian Journal of Botany* 81: 330-337.

Franks SJ, Peterson CJ. 2003. Burial disturbance leads to facilitation among coastal dune plants. *Plant Ecology* 168:13-21.

Gagné J, Houle G. 2001. Facilitation of Leymus mollis by Honckenya peploides on coastal dunes in subartic Quebec, Canadá. *Canadian Journal of Botany* 79: 1327-1331.

Garbin ML, Carrijo TT, Sansevero JBB, Sánchez-Tapia A, Scarano FR. 2012. Subordinate, not dominant, woody species promote the diversity of climbing plants. *Perspectives in Plant Ecology, Evolution and Systematic* 14: 257-265.

Houle G. 1996. No evidence for interspecific interactions between plants in the first stage of succession on coastal dunes in subarctic Quebec, Canada. *Canadian Journal of Botany* 75:902-915.

Joy DA, Young DR. 2002. Promotion of mid-sucessional seedling recruitment and establishment by *Juniperus virginiana* in a coastal environment. *Plant Ecology* 160:125-135.

Maltez-Mouro S, Maestre FT, Freitas H. 2010. Co-occurrence patterns and abiotic stress in sand-dune communities: their relationship varies with spatial scale and the stress estimator. *Acta Oecologica* 36: 80-84.

Martínez ML, Pérez-Maqueo O, Vásquez VM. 2004. Facilitative interactions on coastal dunes in response to seasonal weather fluctuations and benefactor size. *Ecoscience* 11: 390-398.

Muhamed H, Touzard B, Le Bagousse-Pinguet Y, Michalet R. 2013. The role of biotic interactions for the early establishment of oak seedlings in coastal dune forest communities. *Forest Ecology and Management* 297: 67-74.

Oriol G, Rautio P, Heikkinen J, Saravesi K, Kozlov MV, Markkola A. 2010. An ericoid shrub plays a dual role in recruiting both pines and their fungal symbionts along primary succession gradients. *Oikos* 119: 1727-1734.

Santoro R, Jucker T, Carboni M, Acosta ATR. 2012. Patterns of plant community assembly in invaded and non-invaded communities along a natural environmental gradient. *Journal of Vegetation Science* 23: 483-494.

Sternberg M, Yu SL, Kutiel PB. 2004. Soil seed Banks, habitat heterogeneity, and regeneration strategies in a Mediterranean coastal sand dune. *Israel Journal of Plant Science* 52:213-221.

Vallés SM, Fernández JBG, Dellafiore C. 2011. Effects of soil, microclimate and vegetation of the native-invasive *Retama monosperma* (L.) in coastal dunes. *Plant Ecology* 212: 169-179.

Villiers AJ, Rooyen MW, Theron GK. 2001. The role of facilitation in seedling recruitment and survival patterns, in the Strandveld Succulent Karoo, South Africa. *Journal of Arid Environments* 49:809-821.
